# Virtual interaction and visualisation of 3D medical imaging data with VTK and Unity

**DOI:** 10.1049/htl.2018.5064

**Published:** 2018-09-21

**Authors:** Gavin Wheeler, Shujie Deng, Nicolas Toussaint, Kuberan Pushparajah, Julia A. Schnabel, John M. Simpson, Alberto Gomez

**Affiliations:** 1 School of Imaging Sciences & Biomedical Engineering, King's College London, London, UK; 2 Department of Congenital Heart Disease, Evelina London Children's Hospital, London, UK

**Keywords:** augmented reality, data visualisation, rendering (computer graphics), computerised tomography, medical image processing, 3D medical imaging data, virtual reality displays, mixed reality displays, manipulation devices, medical image applications, virtual environments, VTK objects, Unity scene, Unity native plugin, simple Unity application, cardiac magnetic resonance images, current augmented reality/virtual reality display devices, augmented reality displays, interaction devices, Unity widespread support, visualisation toolkit, visualisation capabilities, OpenGL context, VTK volume rendering, thoracic computed tomography, time 3.0 d

## Abstract

The authors present a method to interconnect the Visualisation Toolkit (VTK) and Unity. This integration enables them to exploit the visualisation capabilities of VTK with Unity's widespread support of virtual, augmented, and mixed reality displays, and interaction and manipulation devices, for the development of medical image applications for virtual environments. The proposed method utilises OpenGL context sharing between Unity and VTK to render VTK objects into the Unity scene via a Unity native plugin. The proposed method is demonstrated in a simple Unity application that performs VTK volume rendering to display thoracic computed tomography and cardiac magnetic resonance images. Quantitative measurements of the achieved frame rates show that this approach provides over 90 fps using standard hardware, which is suitable for current augmented reality/virtual reality display devices.

## 1 Introduction

Medical imaging is an invaluable tool in diagnosis and treatment planning. Volumetric images are available from different modalities such as computed tomography (CT), magnetic resonance (MR), or 3D ultrasound (US). Four-dimensional (3D + time) image sets extend this to show function over time, e.g. blood flow or cardiac motion. These data are primarily viewed using 2D screens, which places a limitation on depth perception and understanding of the true 3D nature of the data.

Recently, there has been a significant boost in virtual reality (VR) and augmented reality (AR) displays, mainly in two forms: head mounted and fish tank displays. Head-mounted VR displays are popular as their price has come down to consumer level, e.g. Oculus Rift (www.oculus.com) and HTC Vive (www.vive.com). AR headsets are mostly used by developers, e.g. Meta 2 (www.metavision.com) and Hololens (Microsoft Corp). Fish tank displays include zSpace (www.zspace.com) and Alioscopy (www.alioscopy.com). Progress in display technology enables natural and intuitive interaction in virtual and augmented environments, e.g. gesture and eye tracking, haptic feedback, and so on. These advanced displays and interaction tools are now widely used in the video game industry, and are slowly penetrating into other sectors such as design, marketing, and medical applications.

Arguably, the most widespread AR and VR development environment is Unity (unity3d.com). Unity is primarily used for video game development and its popularity is due to good support, fast prototyping capabilities, and compatibility with most commercially available VR/AR displays and interaction tools. Unity has also been used to develop medical applications, mainly surgery simulators [1]. However, native Unity visualisation capabilities for medical images are somewhat limited to surface rendering of mesh models.

Various visualisation libraries are specifically designed for medical imaging, among which the Visualisation Toolkit (VTK – www.vtk.org) is a de facto standard. VTK is used in many medical imaging software, e.g. Paraview, ITKSnap, MITK and 3D Slicer.

The surface rendering techniques used in recent VR and AR medical visualisation systems built using Unity require a patient-specific polygonal model of the anatomy of interest [1, 2]. Such surface models are typically derived from medical images through segmentation, using manual or semi-automatic methods. In most cases, this involves manual effort, and the time and skill to do this may be significant [3]. Moreover, the segmentation process inherently loses information present in the original volume data. Volume data often do not have precise boundaries, but volume rendering allows the user to interactively tune rendering parameters or apply filters, to achieve the desired appearance. By integrating volume rendering in VR, we remove the potentially erroneous segmentation steps and give the user more flexibility and control.

In this work, we aim to integrate VTK into Unity to bring the medical imaging visualisation features of VTK into interactive virtual environments developed using Unity. Particularly, we describe a method to integrate VTK volume rendering of 3D medical images into a VR Unity scene, and combine the rendered volume with opaque geometry, e.g. sphere landmarks. We focus on creating core technology to enable this and give developers and researchers the ease of use and flexibility of Unity combined with the volume rendering features of VTK.

This Letter is organised as follows. Section 2 briefly summarises the background of Unity and VTK, and the limitations of existing software technology that integrates the two for medical image visualisation. Section 3 elaborates our method for addressing these limitations. Section 4 describes the materials and procedure of a preliminary experiment, and the results are shown in Section 5. Section 6 discusses the results and concludes the Letter.

## 2 Background

### 2.1 Unity

Unity is a cross-platform environment for developing 2D, 3D, VR, and AR video games on many mobile, desktop, and web platforms. Unity supports some of the most popular VR APIs, such as Oculus and OpenVR. Most other headset and 3D display devices are also supported in Unity, often through a manufacturer plugin.

In the medical field Unity has been used to create VR training environments [1, 2], and for scientific and medical visualisation [4]. These studies use surface rendering techniques, which require segmenting a surface from medical images. It is desirable to directly render 3D medical images using volume rendering, which can be implemented in Unity through fragment shaders. Other authors have implemented volume rendering specifically for Unity [5–7]. However, most existing volume rendering technology is available as separate libraries. More interestingly, these libraries incorporate volume rendering interaction features such as cropping, multi-planar reformatting, and transfer functions, which would otherwise need to be implemented for Unity too.

Fortunately, Unity provides a low-level native plugin interface [8] to enable multi-threaded rendering through plugin callbacks. Considering that Unity supports multiple graphics contexts, including Direct3D and OpenGL Core, it is possible to call OpenGL rendering from external plugins and display it directly in Unity.

### 2.2 Visualisation Toolkit

VTK is an open-source C++ library for 3D computer graphics, image processing, and visualisation, aimed particularly at medical imaging visualisation. Crucially, VTK implements OpenGL rendering, recently updated to OpenGL Core, which can be used in external applications. This opens the possibility of OpenGL context sharing between VTK and Unity.

Integrating VR into VTK has attracted great research interest already. For example, adding VR support to the VTK-based medical application MeVisLab [9]. Recent updates to VTK added support for OpenVR and Oculus VR platforms [10], in turn allowing VR rendering to be used with e.g. Paraview. While VTK includes tools for composing scenes and interacting with them, its capabilities are not as advanced as Unity's, and its support of visualisation and interaction platforms, e.g. haptic gloves, is more limited.

Significant effort has gone into VTK's volume rendering capabilities, with a major overhaul in 2014 [11]. Volume rendering functionality includes cropping, composite, and additive blending, maximum and minimum intensity projections, and so on. It has been optimised to run on the GPU in an effort to maximise performance. A target frame rate in frames per second (fps) can be set, with VTK adapting render quality to meet it. Further enhancements have since been made, including an upgrade to OpenGL 3.2 (an OpenGL Core specification), multi-component data support and performance improvements [12].

### 2.3 Related work

An existing approach to integrate VTK in Unity is to generate a virtual scene in VTK, copy the geometry and textures to Unity, and then render them as Unity game objects. A straightforward way to achieve this is to wrap VTK into a C# plugin so that VTK functions can be directly called in Unity scripts. Several C# wrapped VTK tools are available [13, 14]. Activiz.Net is developed by Kitware, Inc., the same company who created VTK. Tamura *et al.* [15] demonstrated a successful case using Activiz.Net in Unity for the implementation of head-mounted display of numerical data.

However, depending on implementation, this approach could be slow due to copy operations, especially as the data get larger and the scene more complex. Copying between the CPU and GPU is generally to be avoided as it causes a GPU stall, and texture copy operations between the CPU and GPU may take of the order of a ms [16]. This delay is significant when our target refresh rate is 90 fps [17], giving ∼11 ms to render each frame. For instance, read-back and upload of two textures in a stereo scene taking 1 ms for each operation uses 8 ms (1 ms × 2 (read-back and upload) × 2 (textures) × 2 (left and right eyes)). A potential solution is to re-implement some of the rendering functionality in the main application. This approach may also increase development workload as it may require re-implementation of the shaders and transfer functions, potentially time consuming and not straightforward, especially in tasks such as volumetric rendering. Inconsistencies in this may result in rendering which does not look as good as the original.

### 2.4 Summary

In summary, Unity provides an ideal development environment for AR/VR applications and advanced 3D interaction. VTK is a medical image visualisation library that provides advanced image display and processing tools including volume rendering. Although some efforts to combine the two exist, previous work sacrifices efficiency for ease of implementation and requires re-implementation of some components. In this Letter, we propose integrating VTK in Unity using a common OpenGL context to keep their native efficiency.

## 3 Methods

To achieve an efficient, close coupling between Unity and VTK, we implement sharing of an OpenGL context between them. The main Unity application manages the OpenGL context, calling VTK at the appropriate time to render directly into the same OpenGL context. The following VTK and Unity features led us to believe this would be successful.

The VTK external rendering module enables VTK rendering in another application's OpenGL context. It is an optional module, selected in the VTK CMake configuration. VTK external rendering is enabled in an application by declaring vtkRenderingExternalModule, and then using the external versions of the camera, renderer and render window. Then VTK does not perform the context setup/tear down it normally would, leaving this to the external application, with the VTK external renderer making its OpenGL calls into the application's OpenGL context.

Unity supports several renderers across numerous platforms. We use the OpenGL renderer to be compatible with VTK. Communication with VTK is also required, and Unity provides a low-level plugin interface [8], where C++ may be used – the native language of VTK. For instance, an available native Unity plugin example [18] uses OpenGL calls to directly manipulate vertex buffers and textures in Unity's OpenGL context, supporting the idea that VTK external rendering in Unity can be achieved.

VTK OpenGL volume rendering is based on the OpenGL 3.2 specification, Unity supports OpenGL 4.5. Both are OpenGL Core specifications. As OpenGL Core is backwards, compatible VTK should not require OpenGL features which Unity does not support.

**Fig. 1:**
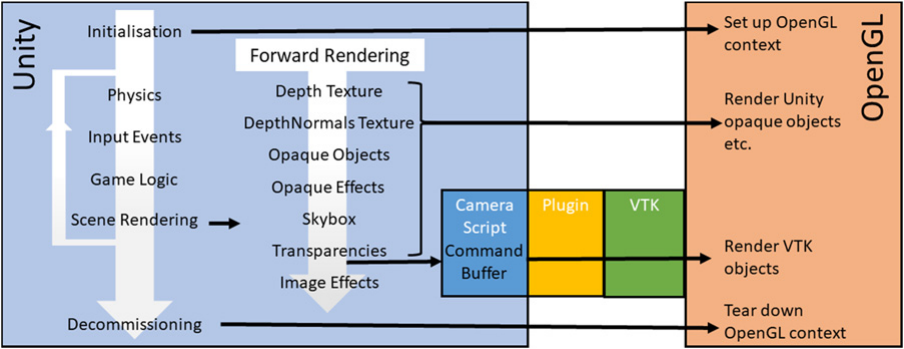
Unity creates, manages, and destroys the OpenGL context. At the appropriate point in the render loop VTK is called to perform volume rendering using the buffers set up by Unity. As the volume is a semi-transparent object, this call is made at the transparency rendering stage

Our proposed architecture to share the OpenGL context is described in Fig. 1. Combining Unity and VTK in this manner offers the possibility of direct and efficient VTK rendering in an OpenGL based Unity application.

### 3.1 Unity plugin implementation

Our plugin acts as the glue to bind VTK to Unity. To ease experimentation and development, this is kept as lightweight as possible, concentrating functionality in the Unity scripts. The plugin connects the vtkCamera to the Unity camera, vtkProp3D derived objects to Unity game objects and synchronises the rendering events.

Unity provides a C API for its event queue and graphics interfaces. This allows plugin development of event handlers and graphics callbacks. A native Unity plugin can also expose static C functions which can be imported into and accessed from Unity C# scripts.

To synchronise the rendering, our plugin registers a graphics callback which calls the VTK Render() method. This callback is associated with the main camera in the Unity scene by adding a command buffer to it in an initialisation script. In Unity these command buffer callbacks are associated with a stage of the rendering loop, e.g. pre- or post-opaque object rendering, for example to perform advanced lighting effects. We add a command buffer to call our plugin's render callback at the transparency render stage. We use this stage as the volume is a semi-transparent object, and so should be rendered after the opaque objects in the scene.

This functionality is adequate to compose a scene in VTK and render it within Unity. However, a bug in the external VTK renderer prevents effective synchronisation of the VTK camera, and a VTK modification was required, described in Sections 3.2 and 3.3. To take advantage of the Unity editor we add functions to allow Unity C# scripts to compose and update the scene. In addition to volume rendering, our plugin makes the following VTK functions available to Unity: data loading (DICOM), add/remove a VTK prop to/from the scene, set a VTK prop's transform (position, rotation, scale), adjust the volume render transfer function, and add a cropping plane to the volume. These functions all directly relate to VTK functionality and are flexible enough for a variety of scenes to be created, manipulated, and explored. Unity and VTK multi-thread many operations, so many of these commands are queued to be safely processed at the render event.

The plugin keeps a std::map of handles linked to the vtkProp3D's pointers in order to manage the scene. When a prop is added to the scene, the plugin returns its handle to be stored as a member variable by the script. The handle is then used by the script to update a prop's position or delete it. The handle is simply an index which is incremented each time a prop is added to the scene.

There are differences between Unity and VTK which we address. First, Unity's unit system is metres, so we scale VTK volume data specified in millimetres to be in metres. Second, Unity uses a left-handed coordinate system while VTK uses a right-handed one. In practice, the camera matrices and actor locations are both reversed so the effects are limited to a *z*-axis flip in the vtkProp3D objects. This affects the volume as a left-right flip, solved by reversing the data along *z* as part of the loading process.

### 3.2 VTK configuration

In the right OpenGL environment, VTK external rendering will work with little effort. However, OpenGL Core pipeline implementations vary as their configuration is determined by the author. Differences between Unity's OpenGL pipeline and VTK's expectations of it have led us to make modifications to VTK for volume rendering and camera updates to work in Unity. These differences are not due to defects in either VTK or Unity. Rather, they are the result of different design decisions made by the two development teams.

By default, the vtkExternalOpenGLRenderer obtains the camera view and projection matrices from OpenGL and sets them in the vtkExternalOpenGLCamera. This works for a legacy OpenGL pipeline, where these matrices are stored in a specified way. However, it does not work for the OpenGL Core pipeline in Unity where the matrices are not stored in the way VTK expects.

We add methods to vtkExternalOpenGLRenderer so the camera view and projection matrices can be set. These are obtained from the Unity camera, and passed in through the plugin. The original code where they are obtained directly from OpenGL was removed. After setting these view and projection matrices in the camera the existing VTK recalculation of the camera up vector and position is performed, required for the camera to work correctly.

**Fig. 2:**
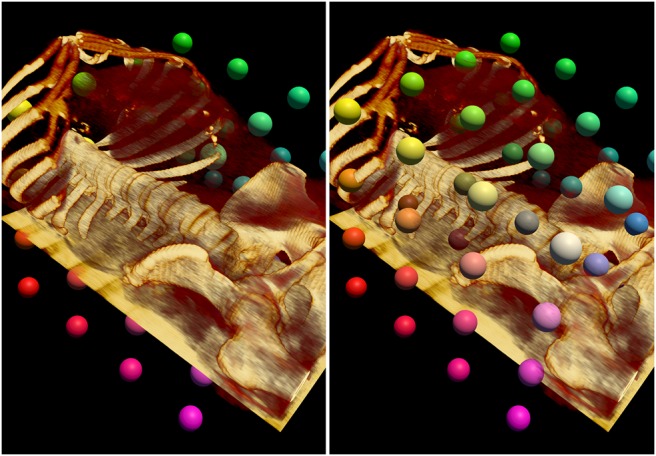
(Left) Before our changes, the polygonal surface rendered landmarks are incorrectly blended with the volume. The markers are often obscured by the volume, when instead the markers should obscure the volume, or be embedded into the volume. (Right) After our changes to VTK, the landmarks correctly obscure and blend in with the volume

These changes are sufficient for VTK opaque surface rendering in Unity, and for volume rendering when none of these surfaces impinges the volume. For volumetric medical data we also require surface rendered objects, e.g. landmarks, within the volume. For the volume and surface rendered objects to be blended correctly VTK volume rendering uses the depth buffer to calculate early ray termination when there is an object inside of the volume [19]. During its render loop, VTK updates depth in the standard GL_BACK_BUFFER, and at the start of the volume rendering copies a depth image from the GL_DEPTH_BUFFER for use in ray termination. Unity uses frame buffer objects (FBO) with textures attached for colour, depth, and so on. As a result, the depth image is not stored in the standard buffer as VTK expects. We address this by obtaining the name of the current FBO's depth texture, and use this as the source of the copy operation. We modified vtkOpenGLGPUVolumeRayCastMapper to copy from the FBO depth texture, requiring an additional method in vtkTextureObject to enable a depth image to be copied from a texture. Fig. 2 illustrates the effect of our changes. The left-hand image illustrates the rendering before our changes, the landmarks are either in-front of the volume, or behind it – but not ‘embedded’ into it. The right-hand image shows the rendering after our changes, the landmarks are correctly ‘embedded’ into the volume render.

We have tested these changes with opaque surface objects embedded within a rendered volume, which yields correctly blended scenes. The opaque objects may be rendered from both Unity and VTK. This configuration works because opaque surface objects are rendered before the volume and write to the depth buffer, which is in turn used by the volume renderer. On the other hand, semi-transparent objects (surface or volume) may not render correctly when combined with another rendered volume due to depth sorting problems. Particularly, rendering multiple volumes will pose depth sorting problems. A potential solution is to render all of the semi-transparent objects in VTK and allow VTK to perform depth sorting and, if necessary, depth peeling. We have not tested other rendering features in VTK, or other OpenGL rendering pipelines, which is left as future work and might require further modifications.

### 3.3 Camera synchronisation

Synchronisation of the VTK camera to the Unity one is performed by a C# script attached to the main camera in the Unity scene. In this script's Start() event a command buffer is added to the camera which will call the plugin's render callback function. No data are passed at this call, so the camera's world-to-camera and projection matrices are obtained from the Unity camera and set in the plugin in the camera's OnPreRender() event.

For a stereoscopic display, e.g. a VR headset, rendering is performed for the left and right eyes. Unity represents a stereo camera as a single camera in its scene making this slightly more complex, rather than as separate left and right eye cameras where stereo rendering is trivial. The OnPreRender() event queries the camera to find out if it is a stereo camera. Then, if it is a stereo camera, if it is the left or right one. From this it obtains the correct view and projection matrices to set in the plugin. The command buffer call to the render event does not indicate if it is being called by the left or right camera, and this cannot easily be ascertained. We therefore make the assumption that the OnPreRender() and render events are called in the same order. In the plugin this is done by pushing the view and projection matrices onto the back of a queue at an OnPreRender() event, then popping them off the front of the queue at the render event, so their order is preserved. Whilst this solution may be improved, our experiments did not show any left-right camera reversal.

### 3.4 Unity scene configuration

Configuring and updating the scene in VTK is also performed with C# scripts. We use the concept of a proxy to enable interaction to be handled by Unity while rendering is performed by VTK. A proxy has a GameObject in the Unity scene, but without any mesh renderer enabled. It may have other active Unity components, e.g. a collider for interaction. A proxy has an active C# script attached to control the creation and update of a prop in VTK. These scripts are structured to fit in with the Unity GameObject lifecycle:

Start() event initialises the VTK prop
Update() sets the VTK prop's transform, updates the window of the transfer function, and so on
Destroy() removes the VTK prop

**Fig. 3:**
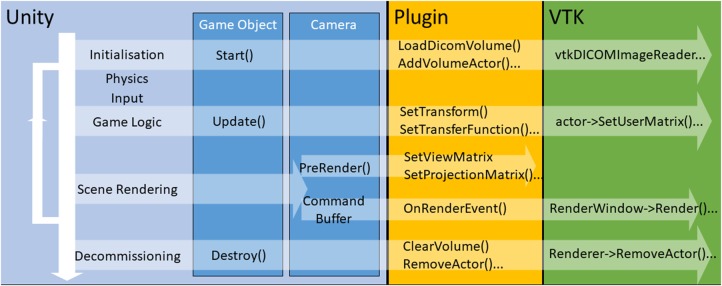
Architecture linking Unity events to VTK functionality via the plugin. For a volume, VTK loads image data and creates a prop during the Unity game object's Start() event. While the Unity application is running, the VTK volume's transform and transfer function are updated in the Update() event. The VTK camera's view and projection matrices are updated by the Unity PreRender() event and the volume is then rendered during the main Unity render pipeline. When the VTK prop is no longer needed the Destroy() event deletes it and unloads the data

For example, our rendered volume is a GameObject to which we attach a collider for controller interaction, and a C# script to initialise, update, and destroy the VTK prop. The scripts actions are illustrated in Fig. 3.

Similarly, a proxy object with a script attached can add and update a surface rendered primitive, e.g. a sphere. Multiple Unity entities can access the plugin functions, e.g. a script can be attached to a controller to alter the transfer function window of the volume.

Volume cropping planes are somewhat more complex as they are entities themselves but also need to be associated with the volume they crop. To associate a cropping plane with a volume, the volume proxy shares its prop handle with the script which creates the cropping plane. This handle is then passed back into the plugin when the cropping plane is added to the scene so that the plugin knows which volume the cropping plane is going to crop.

A cropping plane should move with the volume when the user moves the volume in the scene, but the user should also be able to move the cropping plane independently of the volume. Achieving this offers a good example of Unity's power and flexibility.

**Fig. 4:**
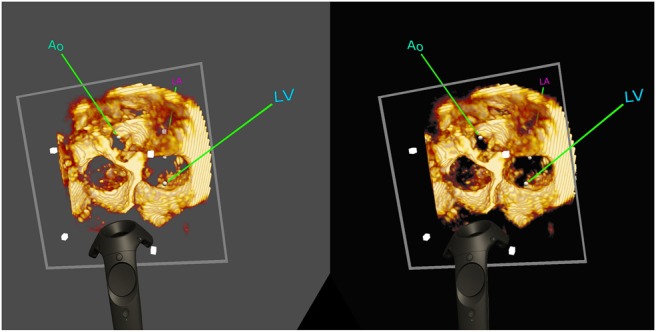
Capture of a stereo rendered scene from an HTC Vive headset. This scene contains an US volume, and its bounding corners are indicated by grey cubes. The volume is cropped by a plane indicated by the grey square outline. Spherical, coloured landmarks have been placed within the volume. The user can pick up and move all of these objects using the standard Vive controller, pictured in the scene

The cropping plane proxy in Unity is created as a child of the volume proxy, so that when the volume moves the cropping plane's position relative to the volume is unchanged. In Unity, this works because an object's transform is relative to its parent. To move the cropping plane independently, its proxy is un-parented from the volume proxy, moved, and then re-parented to the volume proxy. Unity updates the parent–child transform automatically. Fig. 4 shows a US volume cropped with a plane.

Currently, we have not synchronised lighting between Unity and VTK. By default, scenes using the external VTK renderer have no lights. Therefore, props within the VTK scene are unlit.

### 3.5 VR, AR, and interaction in Unity

We have primarily been developing with the HTC Vive and the Unity SteamVR plugin. These are well developed and the combination of controllers and lighthouses gives significant flexibility.

The SteamVR plugin includes a CameraRig prefab, providing a camera which tracks the Vive headset, and tracked representations of the left and right-hand controllers. To use this, we add the CameraRig prefab to the scene and delete the original camera. To enable VTK rendering, we attach our C# script to the Camera (eye) component of the CameraRig. To create interactions using the controllers, we add interaction scripts to the Controller (left) and Controller (right) components, e.g. to pick up and place objects using the trigger, or control the transfer function windowing using the touchpad.

## 4 Materials and experiments

We implemented a Unity plugin and the described VTK modifications which allow volumetric medical data to be loaded and visualised using the VTK volume renderer within a Unity application. In this work, we used Unity 2018.1 and VTK 8.1, under Windows 10 on a standard workstation (Intel Core i5-7600 CPU, NVidia Quadro M4000 8 GB GPU, and 16 GB RAM). We used medical images from two modalities. First, a thoracic CT dataset obtained from the TCGA-SARC dataset (http://cancergenome.nih.gov), and made up of $512 \times 512 \times 100$ voxels ($379 \times 379 \times 500\, {\rm mm}$). Second, a cardiac MR dataset acquired on a healthy volunteer using a Philips Achieva 1.5 T scanner, and made up of $480 \times 480 \times 110$ voxels ($280 \times 280 \times 110\, {\rm mm}$). We chose CT and MR data so we have examples with soft and hard tissue that are relevant for a wide variety of medical applications. These particular volumes were used as their size makes them suitable for volume rendering performance tests.

Rendering performance was measured quantitatively through the rendering frame rate, using the Unity preview Stats overlay to manually read the fps. The volume proxy in Unity was made a child of the SteamVR camera, and the *z*-axis distance between them was adjusted so that the rendered volume filled a different portion of the view. At its closest, the render would mostly fill the viewport. We moved the volume away from the camera to measure the impact on the frame rate. A transfer function where anatomy could be seen clearly – some parts of the render were fully transparent and some parts fully opaque – was manually set, and the volume was the only object in the scene.

Qualitative results are provided as 3D rendered images where opaque landmark spheres are included in the volume, to demonstrate semi-opaque blending.

## 5 Results

Table 1 shows the quantitative results for two imaging modalities. For VR applications, our performance target is 90 fps [17], which is achieved with our proposed method.

**Table 1:** VTK volume rendering performance of a CT and MR volume on the HTC Vive using the Unity editor, with and without a VTK fps target. At the closest distance the volume fills the height of the view, it is then moved away to fill half, and quarter (1/4) of the height

Modality	Vol. Dist.	No fps target	fps target
CT	0.5 m (full)	40–50	70 to >100
MR	0.4 m (full)	55–60	>100
CT	1.0 m (half)	70–80	85 to >100
MR	0.8 m (half)	60–70	>100
CT	2.0 m (1/4)	80–90	90 to >100
MR	1.6 m (1/4)	80–90	>100

VTK allows a performance target to be set in fps and will adapt render quality to maintain this frame rate. This is enabled by setting a frame rate target in vtkExternalOpenGLRenderWindow using the SetDesiredUpdateRate method. The per-frame time for the desired frame rate is then shared between the props in the scene, so as more props are added each one has less render time. For our application, the vtkOpenGLGPUVolumeRayCastMapper adjusts its sampling distance to complete rendering within its allocated frame time if AutoAdjustSampleDistances is enabled.

With no VTK fps target, the frame rate decreases as the volume gets closer to the viewer. This behaviour was expected. As the volume rendering needs to fill more pixels on the display, more rays need to be cast which in turn results in a higher computational cost. When the volume is very close to the viewer, the frame rate can drop to an uncomfortable 40 fps. However, this is the use case VR encourages when we give the user the ability to pick up a volume and examine it. For viewer comfort, the rendering performance needs to be improved.

**Fig. 5:**
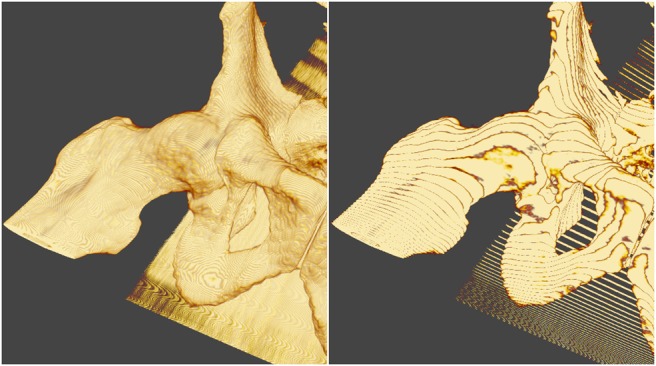
Effect of VTK frame rate target on render quality. (Left) Without a target frame rate quality is maintained but the frame rate can be reduced to an uncomfortable level. (Right) A frame rate target of 100 fps increases viewer comfort, but there can be a visible drop in quality, seen here as an extreme case of a banding effect

Our target frame rate is 90 fps and there are 3 rendered views, headset left and right eyes and the screen preview. Therefore, VTK needs to render the volume 270 times every second. Based on this and allowing for some overhead, we set VTK a 300 fps frame rate target. This significantly increases the final frame rate, often to much higher than 100 fps. However, there is a cost to image quality, with visible banding on the render with a target frame rate (Fig. 5).

Fig. 2 demonstrates that the VTK volume rendering blends correctly with opaque spheres, which are correctly occluded by an MR volume when they are behind a visible portion of it, and in turn occluding the MR volume render when they are in front. Correct cropping of a US volume by an arbitrary plane is shown in Fig. 4.

## 6 Discussion and conclusion

We have successfully integrated VTK volume rendering into Unity. Our method allows opaque surface rendered polygonal objects to be mixed with a VTK volume render. Interactive rendering rates can be achieved, although with some cost to render quality. However, we believe that frame rate should be prioritised so as to avoid possible discomfort. Our initial demonstrations with clinicians have been very positive, so perhaps comfort, interactivity, and intuitiveness outweigh ultimate visual fidelity.

Compared to existing integration methods, e.g. a C# wrapped VTK plugin, our method can achieve ‘what you see in VTK is what you get in Unity’, with improvements of performance and implementation efficiency. The shared OpenGL context avoids the need to copy buffers, the virtual objects are directly rendered and displayed. Moreover, re-implementation of the shaders is also avoided. Specifically, it is more convenient to transplant existing VTK code into Unity with few modifications, although systematic evaluation in the future is required to support this argument.

Our aim was to build a technical platform from which we can develop an intuitive 3D control system, which we have successfully achieved. With Unity and VTK working together the community will have access to a platform where immersive VR/AR medical imaging applications can be quickly built and investigated.

Future work will include further improvements in the performance of our software prototype. For this improvement work we will use more sophisticated tools for performance analysis, e.g. FCAT, and produce more complex scenes with more objects. The optimal trade-off between frame rate and render quality should also be investigated. Other improvements in the system will include the utilisation of transparent objects, lit VTK rendering, and increased VTK volume rendering functionality. We aim to make this work publicly available, and perhaps to integrate it into VTK.

Support for platforms more mobile than our existing desktop Windows/x86 implementation also offers interesting possibilities. For instance, Hololens development is theoretically possible – Unity supports Hololens development, and VTK could be compiled for the Hololens's Universal Windows Platform. This would give much more freedom to users, provided the hardware is powerful enough run volume rendering with adequate visual quality.

## 7 Funding and declaration of interests

This work was supported by the NIHR i4i funded 3D Heart project [II-LA-0716-20001]. This work was also supported by the Wellcome/EPSRC Centre for Medical Engineering [WT 203148/Z/16/Z]. The research was funded/supported by the National Institute for Health Research (NIHR) Biomedical Research Centre based at Guy's and St Thomas’ NHS Foundation Trust and King's College London and supported by the NIHR Clinical Research Facility (CRF) at Guy's and St Thomas. The views expressed are those of the author(s) and not necessarily those of the NHS, the NIHR or the Department of Health. The results shown here are in part based on data generated by the TCGA Research Network: http://cancergenome.nih.gov/.

## 8 Conflict of interest

None declared.
